# Cardiovascular Risk Assessment via Sleep Patterns and ECG-Based Biological Age Estimation

**DOI:** 10.3390/jcm14103339

**Published:** 2025-05-11

**Authors:** Gouthamaan Manimaran, Sadasivan Puthusserypady, Helena Dominguez, Jakob E. Bardram

**Affiliations:** 1 Department of Health Technology, Technical University of Denmark, 2800 Copenhagen, Denmark; sapu@dtu.dk (S.P.); jakba@dtu.dk (J.E.B.); 2 Department of Cardiology, Bisperbjerg-Frederiksberg Hospital, 2400 Copenhagen, Denmark; maria.helena.dominguez.vall-lamora.02@regionh.dk

**Keywords:** polysomnography, deep Learning, cardiovascular risk

## Abstract

**Background:** Understanding the intricate relationship between sleep quality and cardiovascular outcomes opens new avenues for risk stratification in cardiovascular diseases (CVDs). This study aims to evaluate the prognostic potential of biological age estimates derived from sleep-stage analysis and nocturnal heart rhythm patterns. **Methods:** Using polysomnographic data from 1149 patients, we extract ECG signals and use an unsupervised clustering approach to generate time-series clusters that capture dynamic fluctuations in heart rhythms. A subsequent deep learning model then estimated individual biological ages from these clusters, revealing associations between the predicted age, sleep patterns, and cardiac function. **Results:** In an independent test set of 736 patients, the predicted biological age was significantly associated with increased mortality (Hazard Ratio [HR] 2.27, *p* < 0.05) and elevated CVD risk (HR 3.56, *p* < 0.001), while models based solely on nocturnal heart rhythms yielded HRs of 2.29 (*p* < 0.05) for all-cause mortality and 3.13 (*p* < 0.01) for CVD risk. **Conclusions:** These findings demonstrate that integrating sleep stage and ECG offers a robust biomarker for cardiovascular risk stratification, paving the way for earlier interventions and more personalized healthcare strategies.

## 1. Introduction

The electrocardiogram (ECG) is a cornerstone in the diagnostic toolkit for cardiovascular diseases (CVDs), the leading cause of all-cause mortality globally [1]. Leveraging the versatility of electrocardiograms (ECGs), researchers have utilized it to ascertain a range of critical details including age, gender [2,3], and historical cardiac events such as myocardial infarction (MI) [4], alongside diagnosing cardiac arrhythmias such as atrial fibrillation (AFib) and supraventricular tachycardia (SVT) [5,6]. Recent advances in artificial intelligence (AI) have broadened what we can do with Electrocardiograms (ECGs). Today, these methods allow us to assess not only traditional heart measures but also conditions like sleep apnea [7], ejection fraction [8], and even body fat percentage [9]. This means that we can gain a more complete picture of a patient’s heart health.

Building on these advances, AI has been leveraged not only to extract traditional parameters from ECG data but also to refine age estimation. While chronological age—a straightforward measure of the time elapsed since birth—remains a common marker, it does not fully capture the complexities of an individual’s health status. This limitation has spurred interest in estimating “biological age”, a more nuanced metric that integrates genetic, lifestyle, nutritional, and comorbid factors [10]. Unlike chronological age, biological age—often referred to as physiological or functional age—offers a dynamic assessment of health by reflecting various dimensions of an individual’s condition. Recent studies have employed diverse methodologies, ranging from evaluations of physical activity levels [11] to analyses of chest radiographs [12], underscoring that these estimates may vary according to the specific health aspects they capture [13]. Studies have also correlated the estimated biological age with clinical outcomes. Cole et al. [14] predicted biological age from brain magnetic resonance imaging (MRI) and showed that the deviations of predicted age are linked to cognitive impairment. Mortality has also shown to be associated with ECG-based age estimation [15]. However, these studies only examine a limited aspect of an individual’s health by utilizing a single modality (e.g., ECG, MRI, X-ray, etc.).

It is important to recognize that biological age estimates derived from different methodologies may not align exactly, as each approach emphasizes distinct aspects of health based on its data source. For instance, biological age estimated from electroencephalography (EEG) primarily reflects neurological conditions and cognitive decline. In contrast, estimates derived from imaging techniques like MRI or chest radiographs highlight structural changes and tissue conditions. Similarly, assessments based on physical activity or metabolic markers provide insights into lifestyle and metabolic health. Each method offers a unique perspective on the aging process, suggesting that biological age should not be viewed as a single universal measurement. Rather, it serves as an indicator of particular health dimensions and aging pathways, collectively contributing to a more comprehensive understanding of an individual’s overall health status.

In this study, we estimate the biological age by examining the interplay between sleep stages and heart activity as captured by ECG signals, which can say a lot about cardiovascular health in relation to sleep physiology. Our central hypothesis is that subtle anomalies in heart behavior during sleep may serve as early indicators of cardiac dysfunction. To address this, we propose a dual-level approach to infer biological age—an indicator that may better reflect cardiovascular health—by integrating two distinct dimensions of sleep behavior:

1. Sleep stages: Analysis of distinct sleep stages, including rapid eye movement (REM) sleep, light sleep, and deep sleep, which are critical for restorative processes and may reveal disruptions associated with cardiac risk.
2. Heart activity: Extraction and interpretation of heart activity patterns from ECG data using unsupervised clustering techniques, thereby capturing variations in heart dynamics during sleep.

This combined methodology not only enhances our ability to estimate biological age but also establishes a novel, data-driven link between sleep behavior and cardiovascular disease (CVD) risk, potentially paving the way for earlier and more targeted intervention strategies in cardiovascular care.

Our contributions can be summarized as follows:

- We propose a novel framework that uses self-supervised learning and extracts temporal features from ECG signals and employs unsupervised clustering to delineate distinct cardiac states during sleep.
- We introduce a dual-level approach that integrates detailed sleep-stage information (including rapid eye movement (REM), light, and deep sleep) with ECG patterns to estimate biological age—a metric that more accurately reflects cardiovascular health compared to chronological age.
- We validate our methodology on 736 patients in the Sleep Heart Health Study dataset, demonstrating that the predicted biological age is significantly associated with increased risks of cardiovascular diseases and all-cause mortality.

## 2. Materials and Methods

This section outlines the methodology used to examine the relationship between sleep patterns and heart behavior, centering on the derivation of a biological age measure and its association with cardiovascular risk. Our analysis relied on the Sleep Heart Health Study (SHHS) dataset [16], which offers electrocardiogram (ECG) data containing the full span of a night’s sleep from the moment of falling asleep to awakening. This dataset presents various sleep stages, including REM cycles and light and deep sleep phase and instances where patients wake up momentarily, furnishing a robust ground truth for our exploration.

### 2.1. Dataset

The Sleep Heart Health Study dataset [16] is a multi-center longitudinal cohort study for determining cardiovascular and other sleep-disordered breathing. A total of 6411 men and women aged above 40 years old were enrolled between 1 November 1995 and 31 January 1998. There were two recordings—one at the baseline visit and another 3 years later. We use data from only the first baseline visit, which consists of 5802 participants. Polysomnographic data including but not limited to 1 lead electrocardiogram (ECG), electroencephalography (EEG), and blood pressure was recorded at the times during the individual’s sleep period. Cardiovascular outcome data were monitored between baseline and 2011.

We utilized relevant recordings from this dataset, including single-lead ECG data (sampled at 125 Hz, corresponding to lead II in standard 12-lead ECGs), annotated sleep-stage cycles, patient demographics (age and sex), follow-up cardiovascular outcomes, and a medical history of conditions such as diabetes and hypertension. The ECG recording duration varied across subjects, encompassing the entire duration of each individual’s sleep period. All ECG signals were standardized before processing them using the self-supervised model.

In our study, we define a cardiovascular outcome as when an individual experiences one or more of the following: stroke, chronic heart failure, and myocardial infarction (MI). We also only consider patients who do not have any of the mentioned cardiovascular diseases at baseline and assign them as high risk if they experience any of the mentioned outcomes within 5 years of the baseline measurement.

Out of the 5802 study participants assessed at baseline, 736 were free of cardiovascular disease (CVD) at enrolment but experienced a CVD event within five years. We treated these individuals as the *high-risk* cohort and split them evenly: 368 cases were used for developing the age estimation model, and the remaining 368 were held out for the final evaluation. Next, we identified 3149 participants who remained CVD-free at both baseline and the 5-year follow-up. From this *low-risk* pool, we carried out the following:

(i) Randomly selected 368 subjects to match the high-risk hold-out set, giving a balanced evaluation cohort of 736 individuals (368 high-risk + 368 low-risk);
(ii) Reserved 2000 subjects solely for learning the unsupervised heart cluster vectors (Section 2.2.1);
(iii) Retained the remaining 781 subjects for the age estimation model (Section 2.2.2).

Together, the 781 low-risk participants and 368 high-risk development cases formed a 1149-subject dataset for training and tuning the age-estimation model. This dataset was stratified by risk status and divided 70%/30% into training and validation sets, preserving equal high-/low-risk proportions in both partitions.

#### 2.1.1. Ethics Statement

The SHHS is a large, multi-center, community-based, prospective cohort study that sought to determine the cardiovascular and other consequences of sleep-disordered breathing (ClinicalTrials.gov Identifier: NCT00005275). The study was performed in accordance with the Helsinki Declaration, and each participant provided written informed consent. The current project was approved in April 2020 by the Ethics Committee of National Center of Neurology and Psychiatry (project number: A2020-012). All analyzed data are publicly available (https://sleepdata.org/, accessed on 5 February 2024).

#### 2.1.2. AI Statement

During the preparation of this manuscript, the authors used large language models, specifically GPT-4 for the purpose of language editing and spell-checking. The authors reviewed and edited the content as needed and take full responsibility for the content of the publication.

### 2.2. Workflow for Sleep-Related Biological Age Estimation

**Figure 1:**
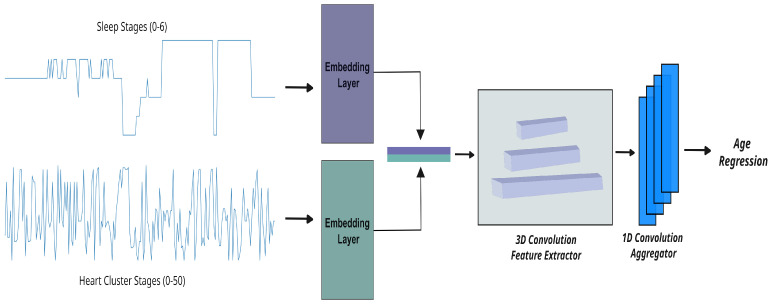
Age regression model architecture: Time-series data from nocturnal cardiac clusters and sleep-stage sequences are first embedded into a learnable space. These embeddings are processed through parallel convolutional blocks with varying kernel sizes to capture multi-scale temporal patterns. The resulting features are refined by neural network layers, enabling the model to predict biological age as a continuous value.

Figure 1 provides an overview of our integrated workflow for predicting sleep-related biological age. This process begins with the transformation of raw ECG signals into *heart cluster vectors* that capture the dynamic patterns of nocturnal cardiac activity, and it progresses through the integration of these vectors with detailed sleep-stage data. In the sections that follow, we describe each component of our methodology in detail, starting with the construction of the heart cluster vectors.

#### 2.2.1. Building the Heart Cluster Vectors

Reading an ECG that spans nearly eight hours (i.e., an entire sleep period) and capturing its intricacies is not a straightforward task. Given the length of the signal, it is impractical to feed the entire raw ECG into an end-to-end AI algorithm for age prediction. Consequently, we convert these raw ECGs into *cardiac states*. Each cardiac state aggregates rhythms with similar waveforms, while distinguishing those that differ. For example, atrial fibrillation and atrial flutter might be grouped into a single cardiac state, whereas ventricular fibrillation would be assigned a distinct state. Furthermore, we can control the granularity of these differentiations by adjusting the number of cardiac states—increasing this number could allow for separate states for atrial fibrillation and atrial flutter.

**Figure 2:**
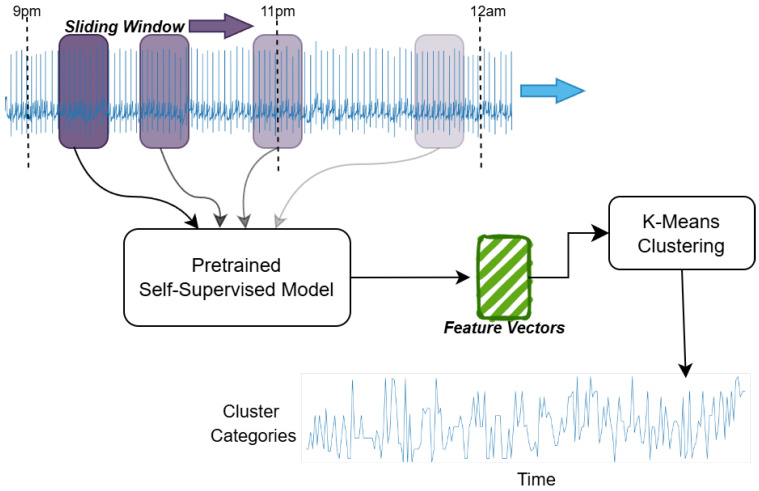
Construction of the heart cluster vectors: ECG signals are segmented using a sliding window and processed through a self-supervised learning framework, resulting in compact feature vectors representing cardiac activity. These vectors are then clustered using K-means clustering to identify distinct patterns of cardiac behavior. Finally, a temporal vector reflecting the sequence of clustered states is constructed, capturing the dynamic changes in heart rhythms across the entire sleep period.

The process of how we convert the ECG into cardiac states is shown in Figure 2 and is explained below.

**Data acquisition and preprocessing**: At the foundation of our analytical pipeline is the preprocessing of the raw ECG signals captured in the SHHS dataset. Our strategy was to convert the long-term ECG signals into a smaller time-series vector that explains the cardiac behavior over an entire night’s sleep. To this end, we split the continuous ECG recordings into non-overlapping segments, each with a duration of 10 s, after which we applied a bandpass filter of order 2 (0.5–40 Hz) and performed the various stages detailed below.

**Self-supervised modeling**: In this work, we train our self-supervised model on a separate database, which is the PhysioNet 2020 Challenge [17,18]. This step is only used to learn ECG representations without explicit labels, like learning patterns of music by repeatedly listening to songs without knowing their genres. Although the details of the self-supervised work is out of the scope of this paper, we introduce the methodology behind training this algorithm to make this paper self-contained. More details about this implementation can be found in our previous work [19].

The input signal is randomly masked by 50%, and its inverse mask is also applied to the same signal. These two signals are trained in a non-contrastive manner using the cosine similarity loss. Along with this branch, the first masked signal is also reconstructed to its original signal to learn the finer details missing inside the signal without the supervision of the second signal. These two paths are trained simultaneously for 25 K epochs. Our approach achieves an 11% improvement in area under the curve (AUC) performance compared to the widely used self-supervised learning framework, Bootstrap Your Own Latent (BYOL), with our method obtaining an AUC of 0.79 versus 0.68 for BYOL.

**Feature vector construction**: We then construct a rich feature vector using the self-supervised model mentioned above to transform each 10-s ECG segment into *feature vectors*. This simple representation captures the key patterns of heart activity, reducing the data’s complexity while preserving important physiological signals for further analysis.

**K-means clustering and hyperparameter tuning**: We applied the K-means clustering algorithm [20] to these feature vectors to group similar patterns of heart activity. After testing different values for the number of clusters, we settled on K = 50, as this value provided a good balance between capturing detailed patterns and maintaining computational efficiency.

**Heart cluster vector construction**: After clustering the ECG features, we construct a vector to capture the evolution of heart activity throughout the night for both training and test cohorts. Each vector reflects the sequence of 50 distinct cardiac states—each state corresponding to a 10 s segment—as the heart transitions through different sleep stages. We call this vector the **heart cluster vector**.

We then visualized this vector alongside the sleep-stage data, providing a clear and integrated view of the nocturnal cardiac dynamics and their relationship to sleep. This heart cluster vector sets the foundation for a deeper exploration of the interplay between sleep patterns and cardiovascular health.

#### 2.2.2. Age Regression Track

To estimate sleep-related biological age, we developed three experimental setups that leverage different aspects of the data derived in the previous sections:

1. **Heart cluster model:** This model only uses the heart cluster vector obtained from the K-means clustering of ECG features (see Section 2.2.1). The vector, which encodes the sequence of 50 distinct cardiac states over the night, is input into a regression model to estimate biological age based on nocturnal heart dynamics.
2. **Sleep-stage model:** This configuration relies solely on the sleep-stage vector—a detailed record of an individual’s sleep stages (ranging from 0 to 6) based on ground truth annotations. While these sleep stages are directly provided in the dataset, they can also be estimated via electroencephalography (EEG) or ECG data [21,22]. This model assesses biological age from the perspective of sleep architecture and its transitions.
3. **Cluster** × **Sleep model:** In this combined approach, we integrate both the heart cluster vector and the sleep-stage vector. By correlating the temporal patterns in cardiac activity with the progression of sleep stages, this model aims to capture the interplay between heart dynamics and sleep behavior. The fused data are then fed into a regression framework, providing a more comprehensive estimate of biological age that reflects cardiovascular risk more accurately.

Each setup is evaluated within a regression framework, comparing the estimated biological age with chronological age and correlating these differences with cardiovascular outcomes. This multifaceted approach enables us to determine which aspects of sleep and cardiac behavior contribute most significantly to the stratification of cardiovascular risk.

Our methodology for computing the biological age in these three experimental setups is detailed below.

**Mapping time-series data to learnable projections:** Our raw data, presented as a categorical time series, are first transformed into a learnable space using embedding layers. These layers map the inputs to a fixed-dimensional space (with N=6), enabling the model to uncover complex patterns in the sequential data.

**Transforming vectors for deep insights:** Once mapped, the vectors are further processed to suit the analytical needs of our neural network. For the heart cluster and sleep-stage models (setups one and two), we apply 2D convolutions on inputs of shape B×T×N, where *B* is the batch size and *T* is the number of time steps. In contrast, the combined model (setup three) merges the heart cluster vector and sleep-stage vector into a tensor of shape B×2×T×N, which is then analyzed using 3D convolutions. To capture both short- and long-term dependencies, we employ three parallel convolutional blocks with kernel sizes of 5, 15, and 31, applying appropriate padding to ensure consistent dimensions throughout. This multi-scale convolutional approach is critical for extracting rich, hierarchical features necessary for accurately predicting biological age.

**Fine-tuning and regression analysis**: In the final stage, the refined vectors are passed through a shallow 1D convolutional network composed of four layers, each progressively enhancing the extracted features. This network concludes with an adaptive pooling layer that adjusts to the varying lengths of the input sequences, ensuring a consistent output size regardless of the original temporal dimensions. Importantly, we approach age prediction as a regression problem—predicting a continuous value—rather than as a classification task. This strategy allows us to capture the subtle and complex patterns within the rhythmic data, ultimately leading to more precise estimates of biological age.

## 3. Results

**Figure 3:**
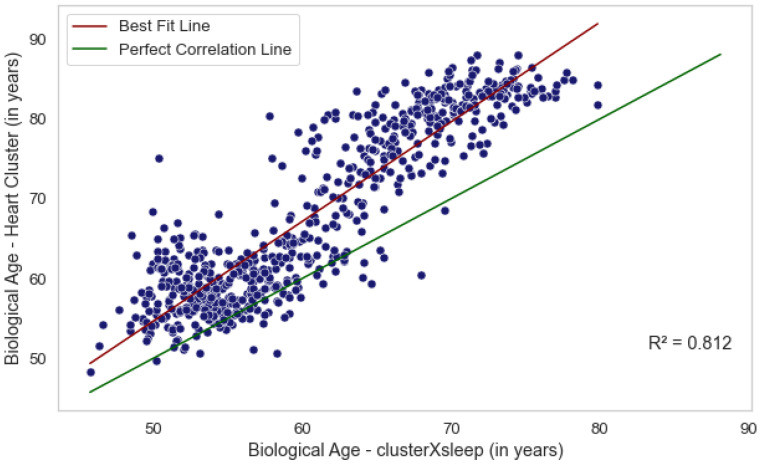
Correlation plot between the biological age predicted by the heart cluster model and the clusterXsleep model showing the high correlation between the two predictions.

We evaluated three methods for estimating biological age from sleep and cardiac data, each capturing a distinct aspect of an individual’s health. Age estimation based solely on sleep-stage data reflects the quality and efficiency of an individual’s sleep cycle, whereas estimations from the heart cluster vector capture nocturnal cardiac activity. Although these approaches derive from different biological mechanisms, our analysis (Figure 3) shows that they are not independent. In particular, the ages predicted by the combined *Cluster × Sleep* model and the *heart cluster* model exhibit moderate correlations with chronological age ($R^{2}=0.295$, *p* = 7.2 × $10^{-58}$ and $R^{2}=0.305$, *p* = 4.5 × $10^{-60}$, respectively) and are also correlated with each other ($R^{2}=0.812$, *p* = 6.5 × $10^{-71}$). The *sleep-stage* model does not show a significant correlation. These findings suggest that integrating heart activity with sleep data provides a more comprehensive assessment of biological age, potentially offering deeper insights into cardiovascular health.

**Figure 4:**
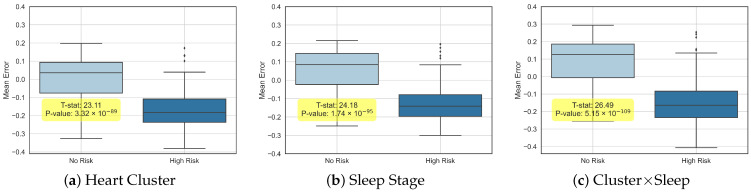
Mean difference across groups for all three experiments for cardiovascular diseases (CVDs).

To further elucidate the distinctions and correlations among the estimated ages, we partitioned the participant cohort into two groups: “No Risk” and “High Risk” (for cardiovascular diseases (CVDs)). Figure 4 presents box plots that illustrate the differences between each individual’s chronological age and the predicted ages from all three experimental setups, highlighting both the variability and complementary insights provided by our models.

In each of the three experiments, we quantified the mean difference between the predicted and chronological ages, along with Hazard Ratios (HRs), for both all-cause mortality and CVD risk (see Table 1). It is important to note that the comprehensive CVD risk measure includes a range of heart-related complications—such as strokes and heart failures—as detailed in the SHHS database [16].

### 3.1. Analysis of Specific Cardiovascular Outcomes

**Figure 5:**
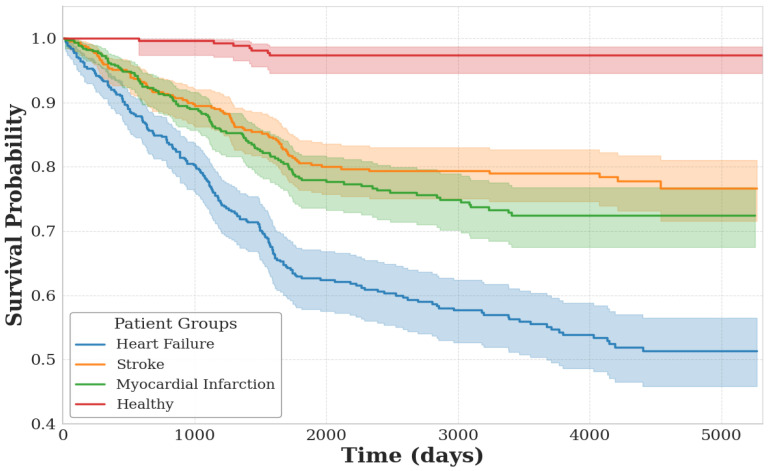
Kaplan–Meier survival curves illustrating the probability of survival over time for individuals who developed heart failure, stroke, or myocardial infarction within the 5-year follow-up period. The healthy cohort is shown as a reference group. Shaded regions represent 95% confidence intervals.

To gain deeper insight into the relationship between aging and cardiovascular risk, we subdivided the broad cardiovascular disease (CVD) category into three specific outcomes: heart failure, stroke, and myocardial infarction. In this analysis, we used the same healthy cohort but defined the risk group as those who were free of these conditions at baseline yet developed at least one of them over a five-year follow-up period. Table 2 presents the Hazard Ratios—adjusted for age and gender—based on the predicted biological age for these subcategories. Heart failure shows the highest risk, followed by myocardial infarction, while stroke risk remains statistically insignificant (high *p*-value). One explanation may be that stroke can either be cardioembolic (originating from a clot in the heart) or thrombotic (originating from a clot in the brain), and in the latter case, ECG readings may not capture any relevant markers. Figure 5 illustrates the corresponding survival curves for these three subcategories, where we can see a much lower survival probability for heart failure.

### 3.2. Comparison with Framingham Risk Variables

To evaluate the clinical utility of our derived biological age metric, we compared its performance to the established Framingham Risk variables [23,24]. The Framingham Risk score—which employs factors such as age, sex, race, total cholesterol, HDL, systolic blood pressure, hypertensive treatment, smoking status, and history of diabetes—is widely used to predict the 10-year cardiovascular disease (CVD) outcome.

**Figure 6:**
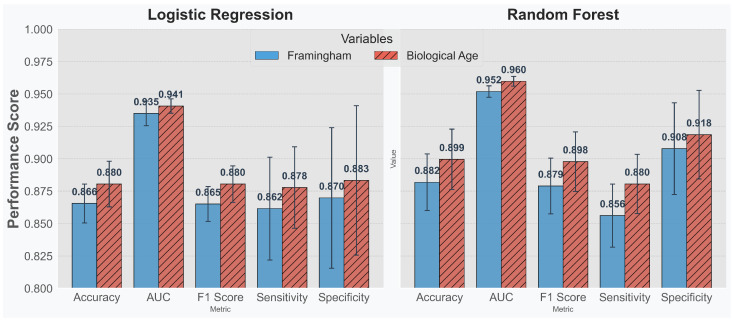
Performance comparison of cardiovascular disease (CVD) risk classification models. The figure demonstrates how the addition of the biological age estimate to the traditional Framingham risk factors leads to improved metrics in both logistic regression and random forest classifier models.

We designed two experimental setups. In the first, logistic regression and a random forest classifier were applied solely to the Framingham variables to predict the 10-year cardiovascular disease (CVD) risk. In the second setup, we augmented the Framingham variables with our estimated biological age to assess whether its inclusion improves prediction performance. Using both a linear model (logistic regression) and a non-linear model (random forest) allows us to evaluate the impact of the additional biological age metric across different types of variable combinations. Figure 6 shows that, across all evaluated metrics, incorporating biological age leads to enhanced performance in both models. This finding suggests that biological age supplements the Framingham variables effectively—improving overall risk stratification rather than simply serving as an ancillary measure.

## 4. Discussion

In this research, we model and assess the deeper correlations between nocturnal heart activity and sleep patterns with the goal of innovating the current methods of stratifying cardiovascular risk. Leveraging the rich data from the SHHS [16] database, we can extract meaningful insights, painting a clearer picture of an individual’s health landscape through our novel age-estimation techniques.

While our approach derives 50 distinct “cardiac states” using unsupervised clustering without pre-defined clinical labels, these states are highly useful for detecting subtle anomalies that may not yet be clinically established. Importantly, when an interesting state is identified, its interpretation is straightforward—clinicians can directly examine the underlying ECG parameters to understand the anomaly. This combination of data-driven detection and ease of clinical interpretation paves the way for integrating these markers into conventional cardiovascular risk assessments.

Integrating both the cardiac states (ECG) and sleep-stage vectors offers a distinct advantage over relying solely on ECG data. While the heart cluster vector effectively captures nocturnal cardiac dynamics, incorporating sleep-stage information provides a richer context by linking these dynamics to the sunderlying sleep architecture. This dual-modality approach not only uncovers a wealth of information embedded in nocturnal biological processes but also enhances our ability to detect subtle, clinically relevant deviations that might be overlooked in an isolated analysis. Furthermore, as evidenced in Table 1, the combined model improves cardiovascular risk stratification—yielding higher Hazard Ratios—while maintaining comparable performance in predicting all-cause mortality relative to the heart cluster model alone. Clinically, this integrated strategy contributes to a more holistic assessment of patient health, facilitating the earlier identification of individuals at risk for adverse cardiovascular events and supporting personalized intervention strategies. Importantly, the observed distinction between predicted biological age and chronological age underscores the value of individualized health metrics, offering a unique perspective that leads to a more rounded understanding of each individual’s health and potential risks. We also believe that this work is only a step towards building the true measure of biological age—one that not only encompasses ECG activity during sleep but also integrates multiple predicted ages from EEG, ECG during activity, MRI, and other modalities reflecting critical body functions to achieve a more holistic representation of overall health. In this envisioned framework, the overall biological age could be conceived as a weighted sum of the individual modality-based predicted ages, or even defined by the minimum value, reflecting the adage that the team is only as strong as its weakest player.

Although our regression models exhibit a substantial mean error (Table 1), a zero error would imply that the model’s predictions simply mirror chronological age, offering no additional insight. In contrast, a meaningful divergence between predicted and chronological ages is essential for developing a metric that effectively evaluates cardiovascular risk beyond the limitations of conventional age measures.

Therefore, while the mean deviation of our regression model serves as a useful performance metric, its primary significance lies in the gap it reveals between predicted and chronological ages. This discrepancy provides critical insights into cardiovascular risk factors that extend beyond what is captured by chronological age alone. By focusing on this difference, our approach supports a more holistic and nuanced assessment of risk, leveraging a richer set of physiological data to inform personalized healthcare strategies.

While the correlations found in this study denote a significant step forward in personalized healthcare, it is pertinent to find explainable causes for the differences in the two ages. Although it stands as a limitation in the current study, it opens avenues for further research to refine these models, perhaps by introducing more variables or leveraging more advanced machine learning algorithms to improve predictive accuracy.

Our current pipeline applies a 0.5–40 Hz bandpass filter and fixed 10 s ECG segments before self-supervised representation learning. Alternative design choices—e.g., sub-5 s windows to capture beat-level dynamics, stronger noise suppression, or data-augmentation methods such as random resampling and simulated baseline wander—could reshape the heart cluster vocabulary and modulate both age-prediction and risk-stratification accuracy. Quantifying how these preprocessing hyperparameters influence the learned embedding space, ideally through systematic ablation studies, is therefore an important next step. Equally critical is external validation: Although the SHHS cohort [16] spans multiple centers, it represents a late-1990s, North American sleep-lab population. Replicating—and, if necessary, recalibrating—the proposed sleep-ECG biological age metric on contemporary, ambulatory, or in-home recordings from more ethnically and clinically diverse populations will be essential before routine clinical deployment.

## 5. Conclusions

This study demonstrates that a comprehensive analysis of sleep-stage and ECG-derived heart rhythm data can yield a robust biomarker for cardiovascular risk stratification. By leveraging an AI framework to estimate biological age, we revealed a meaningful divergence from chronological age that captures subtle, underlying health dynamics. The metric derived (biological age) offers a more nuanced assessment of cardiovascular risk, moving beyond conventional age-based evaluations. Our findings pave the way for earlier and more targeted interventions, aligning with a personalized approach to healthcare. Ultimately, refining this methodology could establish it as a predictive tool in clinical practice—redefining age as a dynamic narrative reflective of an individual’s cardiovascular health rather than a static number.

## Figures and Tables

**Table 1:** Summary of risk stratification of all three experiments. Values are calculated over 10 different seeds and are shown as mean (standard deviation). We show that age computed using the correlation of sleep and heart rhythms gives the most statistically significant result with the highest Hazard Ratio (shown in bold).

	Heart Cluster		Sleep Stages		Cluster × Sleep	
	**Hazard Ratio**	***p*** **-Value**	**Hazard Ratio**	***p*** **-Value**	**Hazard Ratio**	***p*** **-Value**
**Total CVD Risk**	3.13 (0.16)	0.01 (0.04)	1.58 (0.17)	0.13 (0.22)	**3.56 (0.11)**	3.2 × $10^{-4}$ (9.6 × $10^{-4}$)
**All-Cause Mortality**	2.29 (0.05)	0.02 (0.02)	2.23 (0.08)	0.02 (0.04)	2.27 (0.08)	0.02 (0.04)
**Mean Error (in years)**	14.3 (1.2)		12.4 (0.4)		15.1 (1.3)	

**Table 2:** Hazard Ratios for specific cardiovascular outcomes.

Outcome	Hazard Ratio	95% CI	*p*-Value
Heart Failure	5.16	[1.91, 13.95]	<0.005
Stroke	1.44	[0.46, 4.54]	0.54
Myocardial Infarction	3.83	[1.48, 9.93]	0.01

## Data Availability

This study uses publicly available datasets that can be found at the following link: https://sleepdata.org/datasets/shhs, accessed on 5 February 2024.

## Abbreviations

The following abbreviations are used in this manuscript:

CVD	Cardiovascular Diseases
NLP	Natural Language Processing
ECG	Electrocardiogram
HR	Hazard Ratio
AFib	Atrial Fibrillation
EEG	Electroencephalography
SVT	Supraventricular Tachycardia
REM	Rapid Eye Movement
MI	Myocardial Infarction
